# STAFFING DIFFERENCES IN NURSING HOME SPECIAL FOCUS FACILITIES AND SPECIAL FOCUS FACILITY CANDIDATES

**DOI:** 10.1093/geroni/igac059.2854

**Published:** 2022-12-20

**Authors:** Annie Rhodes, Faika Zanjani, Thomas Caprio, Tracey Gendron, Leland Waters

**Affiliations:** Virginia Commonwealth University, Richmond, Virginia, United States; Virginia Commonwealth University, Richmond, Virginia, United States; University of Rochester, Rochester, New York, United States; VCU, Richmond, Virginia, United States; Virginia Commonwealth University, Richmond, Virginia, United States

## Abstract

**Introduction:**

Centers for Medicare and Medicaid Services designates “Special Focus Facilities” (SFF), which are nursing homes receiving special oversight for persistent quality problems. “Special Focus Facility Candidates” (SFFc) are nursing homes with quality problems that are designated as candidates to be future SFFs. Recent academic literature has not examined if SFF and SFFc have significant differences in staffing hours per resident day (HPRD).

**Methods:**

Nursing homes that were SFF or SFFc in 2020 were matched to 5-star nursing homes. Monthly staffing averages were aggregated, and two-way ANOVAs with Tukey Post Hoc were conducted to detect level differences in HPRD across the three facility groups. Gross and Case-Mix staffing HPRD were analyzed, along with daily resident census.

**Results:**

The final sample was n=197 SFFc, and n= 50 SFF, which were matched with n=247 5-star nursing homes for a total sample of Nf494. Over 2020, daily census for 5-star nursing homes was lower (M=72.36, SD=52.5) than SFFc (M=93.77, SD=68.34) or SFFs (M=97.88, SD=44.06). There was a significant difference between SFF and SFFc HPRD in Case-Mix Aide F(2, 5748)=187.6, p= < .005, and Registered Nurse (RN), F(2,5748)=323,p=.003 care. There were no significant differences between SFF and SFFc in HPRD Aide F(2,5748)=380,p=.63, Practical Nurse F(2,5748)=1.1, p=.211, Case-Mix Practical Nurse F(2,5748)=19.57,p=.39, Case-Mix RN F(2,5748)=9.51,p=.91 or Total HPRD F(2,5748)=472.6,p=.16, care. Discussion: There is only a significant difference in staffing levels observed between SFF and SFFc for Aide staffing and RN staffing. This information supports researchers and policymakers in delineating the differences and similarities between SFF and SFFcs.

